# Author Correction: Tuning microtubule dynamics to enhance cancer therapy by modulating FER-mediated CRMP2 phosphorylation

**DOI:** 10.1038/s41467-022-31011-1

**Published:** 2022-06-10

**Authors:** Yiyan Zheng, Ritika Sethi, Lingegowda S. Mangala, Charlotte Taylor, Juliet Goldsmith, Ming Wang, Kenta Masuda, Eli M. Carrami, David Mannion, Fabrizio Miranda, Sandra Herrero-Gonzalez, Karin Hellner, Fiona Chen, Abdulkhaliq Alsaadi, Ashwag Albukhari, Donatien Chedom Fotso, Christopher Yau, Dahai Jiang, Sunila Pradeep, Cristian Rodriguez-Aguayo, Gabriel Lopez-Berestein, Stefan Knapp, Nathanael S. Gray, Leticia Campo, Kevin A. Myers, Sunanda Dhar, David Ferguson, Robert C. Bast, Anil K. Sood, Frank von Delft, Ahmed Ashour Ahmed

**Affiliations:** 1 Ovarian Cancer Cell Laboratory, Weatherall Institute of Molecular Medicine, University of Oxford, Headington, Oxford, OX3 9DS UK; 2 Nuffield Department of Obstetrics & Gynaecology, University of Oxford, Women’s Centre, John Radcliffe Hospital, Oxford, OX3 9DU UK; 3 Structural Genomics Consortium, Nuffield Department of Medicine, University of Oxford, Old Road Campus Research Building, Oxford, OX3 7DQ UK; 4 Department of Gynecologic Oncology, The University of Texas MD Anderson Cancer Center, 1515 Holcombe Blvd, Houston, TX 77030 USA; 5 Center for RNAi and Non-Coding RNA, The University of Texas MD Anderson Cancer Center, 1515 Holcombe Blvd, Houston, TX 77030 USA; 6 Biochemistry Department, Faculty of Science, King Abdulaziz University, Jeddah, 21551 Saudi Arabia; 7 Wellcome Trust Centre for Human Genetics and NIHR Biomedical Research Centre, Roosevelt Drive, Oxford, OX3 7BN UK; 8 Department of Statistics, 1 South Parks Road, Oxford, OX1 3TG UK; 9 Department of Experimental Therapeutics, University of Texas MD Anderson Cancer Center, Houston, TX USA; 10 Goethe-University Frankfurt, Institute for Pharmaceutical Chemistry and Buchmann Institute for Life Sciences, Riedberg Campus, Frankfurt am Main, 60438 Germany; 11 Department of Biological Chemistry and Molecular Pharmacology, Harvard Medical School, Boston, MA 02115 USA; 12 Department of Cancer Biology, Dana-Farber Cancer Institute, Boston, MA 02215 USA; 13 Department of Oncology, University of Oxford, Old Road Campus Research Building, Roosevelt Drive, Oxford, OX3 7DQ UK; 14 Department of Histopathology, Oxford University Hospitals, Oxford, OX3 9DU UK; 15 Nuffield Division of Clinical Laboratory Sciences, Radcliffe Department of Medicine University of Oxford, Oxford University Hospitals, Oxford, UK; 16 Diamond Light Source Ltd, Harwell Science and Innovation Campus, Didcot, OX11 0QX UK; 17 Department of Biochemistry, University of Johannesburg, Auckland Park, 2006 South Africa

Correction to: *Nature Communications* 10.1038/s41467-017-02811-7, published online 2 February 2018.

Since the publication of this work, Eli M Carrami has changed their name from Mohammad Karaminejadranjbar. This has now been amended in the HTML and PDF versions of the article.

