# Supplementary material for: Tuning microtubule dynamics to enhance cancer therapy by modulating FER-mediated CRMP2 phosphorylation
Source: Nat Commun. 2018 Feb 2;9:476. doi: 10.1038/s41467-017-02811-7 (PMC5797184; doi:10.1038/s41467-017-02811-7)
Supplement: Supplementary file 1 — Supplementary Information [file 41467_2017_2811_MOESM1_ESM.pdf]

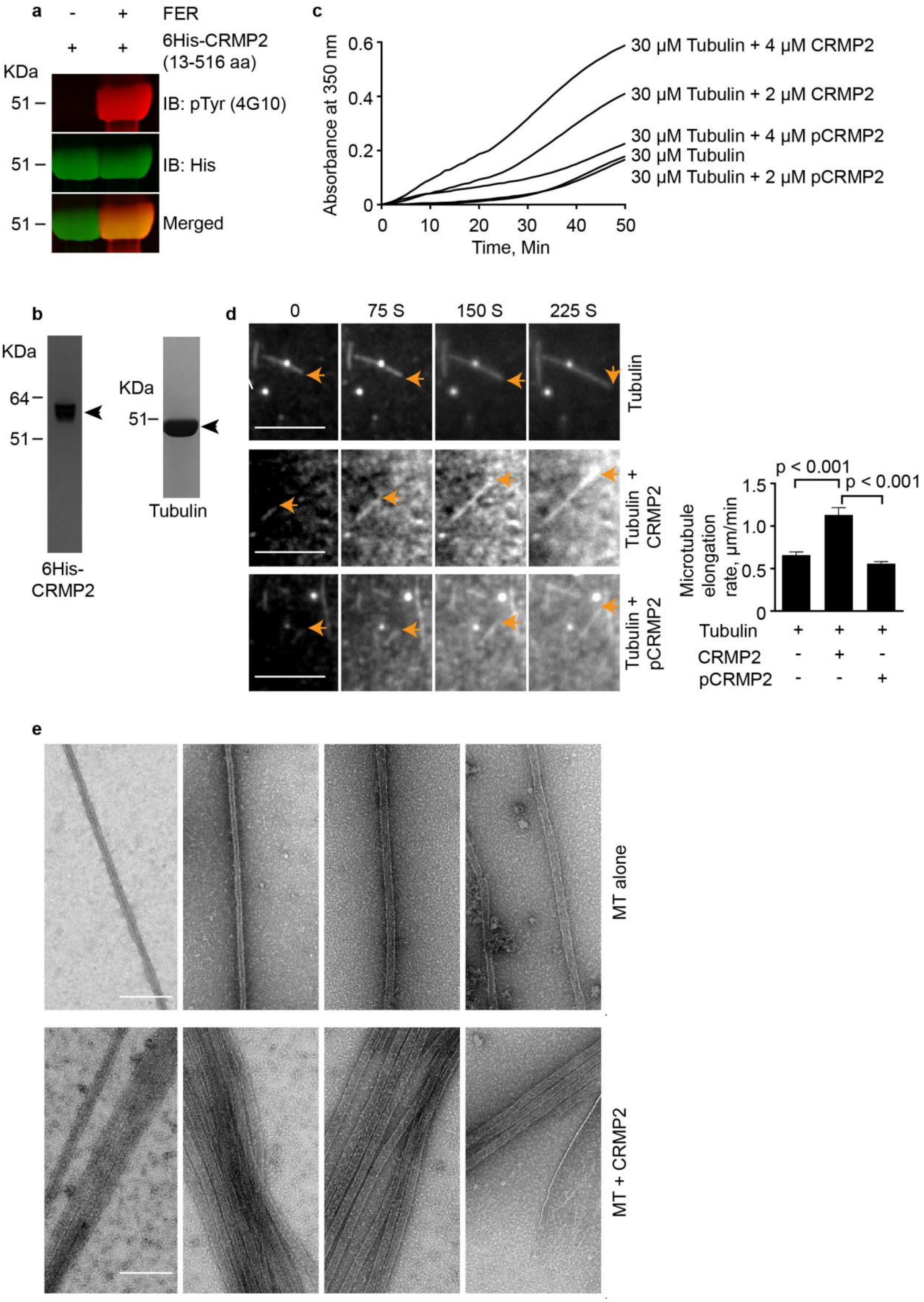

Supplementary Figure 1. (**a**) Recombinant 6His-CRMP2 (residues 13-516) was purified and used for *in vitro* kinase assays in the absence or presence of active GST-FER protein

(residues 521-822). The samples were analyzed by SDS-PAGE, and immunoblotted with the indicated antibody. (**b**) SDS-PAGE and coomassie staining for recombinant 6His-CRMP2, and tubulin which was purified from pig brains. (**c**) Tubulin polymerization was measured by absorbance at 350 nm in the absence or presence of CRMP2 or pCRMP2 (phosphorylated by FER kinase). (**d**) Time lapse of microtubule elongation using total internal reflection fluorescence microscopy (TIRFM). 400 nM GMPCPP-microtubule seeds were incorporated with 10% of rhodamine-labeled microtubules and attached onto glass coverslips that were pre-incubated with anti-α-tubulin antibody. 7.5 μM 50% rhodamine-labeled tubulin was injected into the reaction chamber to initiate microtubule elongation in the absence or presence of CRMP2 or pCRMP2 (phosphorylated by FER kinase). Images were obtained at the indicated time points. The orange arrowheads point to the leading edge of a single microtubule. Bar plots are shown as mean + s.e.m. of microtubule elongation rate, from at least 25 individual microtubules, shown are typical results from at least three independent replicates. Scale bar is 5 μm. See also Supplementary movie 1-3. (**e**) More examples of electron microscopy images of microtubules in the absence or presence of recombinant 6His-CRMP2. Scale bar is 200 nm.

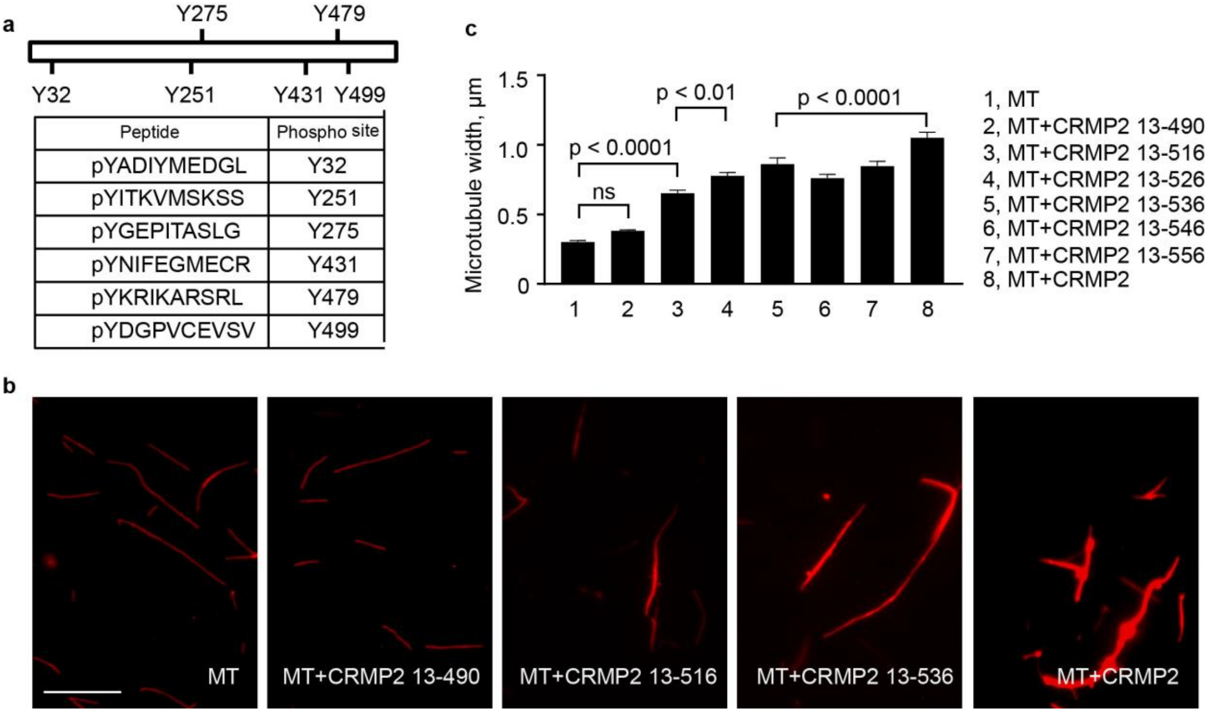

Peptide	Phospho site
pYADIYMEDGL	Y32
pYITKVMSKSS	Y251
pYGEPITASLG	Y275
pYNIFEGMECR	Y431
pYKRIKARSRL	Y479
pYDGPVCEVSV	Y499

Supplementary Figure 2. (**a**) A schematic showing the phosphorylation sites of CRMP2, mediated by FER kinase, that were revealed by Mass Spectrum phospho-mapping. (**b**) The indicated recombinant CRMP2 wild-type or truncated CRMP2 proteins were incubated with paclitaxel-stabilized rhodamine-labeled microtubules for 40 minutes at room temperature. Shown are typical results from at least three independent replicates. Scale bar is 10 µm. (**c**) Bar plots represent the mean + s.e.m. of microtubule width, from at least 150 individual microtubules per condition tested.

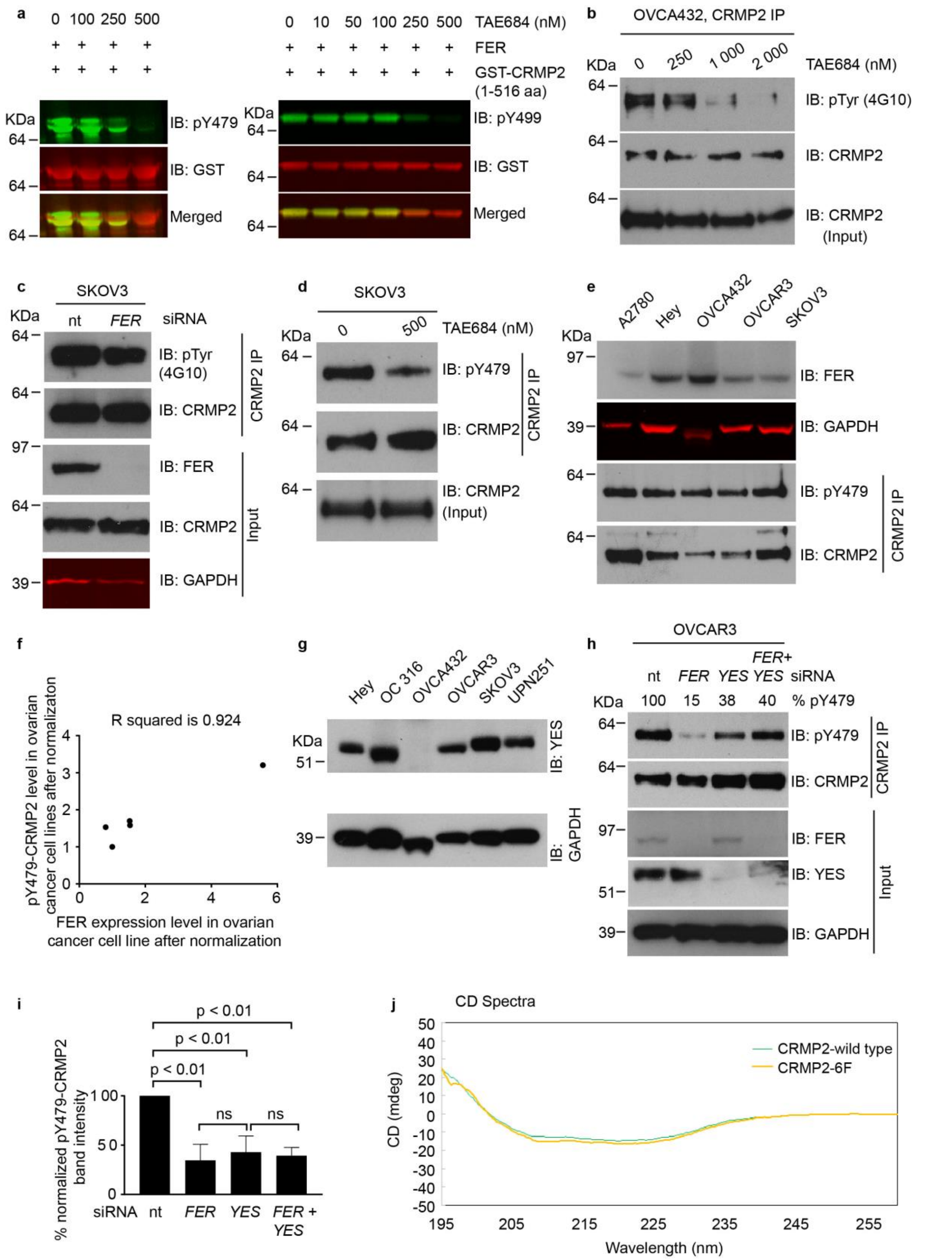

Supplementary Figure 3. (**a**) Recombinant GST-CRMP2 (residues 1-516) was purified and used for *in vitro* kinase assays in the absence or presence of active GST-FER protein (residues 521-822), and incubated with increasing concentrations of the FER inhibitor

TAE684. The samples were analyzed by SDS-PAGE, and immunoblotted using the indicated antibodies. (**b**) Lysates of OVCA432 cells, which were treated with increasing concentrations of TAE684, were immunoprecipitated with anti-CRMP2 antibody. The samples were analyzed by SDS-PAGE, and immunoblotted with the indicated antibodies. 4G10 is a general anti-phosphotyrosine antibody. CRMP2 was loaded as a control for protein input. IB, immunoblot. (**c**) Lysates of SKOV3 cells, which were treated with non-targeting (nt) siRNA or *FER* siRNA, were immunoprecipitated with anti-CRMP2 antibody. The samples were analyzed by SDS-PAGE, and immunoblotted with the indicated antibodies. CRMP2 was loaded as a control for protein input. Also shown are immunoblots that confirm the depletion of FER. (**d**) Lysates of SKOV3 cells, which were treated with or without 500 nM TAE684, were immunoprecipitated with anti-CRMP2 antibody. The samples were analyzed by SDS-PAGE, and immunoblotted with the indicated antibodies. (**e**) Lysates of the indicated ovarian cancer cell lines were immunoprecipitated using an anti-CRMP2 antibody. The samples were analyzed by SDS-PAGE, and immunoblotted with the indicated antibodies. Shown are typical results from three independent replicates. (**f**) Correlation analysis of pY479-CRMP2 and FER protein expression level in multiple ovarian cancer cell lines, including A2780, Hey, OVCA432, OVCAR3, and SKOV3 cell lines. pY479-CRMP2 level was determined by (pY479-CRMP2) / (total CRMP2) in individual cell lines. (**g**) Western immunoblot (IB) analysis for YES kinase expression in multiple ovarian cancer cell lines using the indicated antibodies. (**h**) Lysates of the ovarian cancer cell line OVCAR3, which was treated with the indicated siRNA, were immunoprecipitated with anti-CRMP2 antibody. The samples were analyzed by SDS-PAGE, and immunoblotted with the indicated antibodies; nt siRNA was non-targeting siRNA. Shown are typical results from three independent replicates. (**i**) Quantification analysis of pY479-CRMP2 in OVCAR3 cells treated with the indicated siRNAs. Bar plots represent the mean + s.e.m. of pY479-CRMP2 level from three replicates. (**j**) CD Spectra comparison of full-length CRMP2 wild-type and CRMP2-6F: Spectra are similar indicating similar secondary structures.

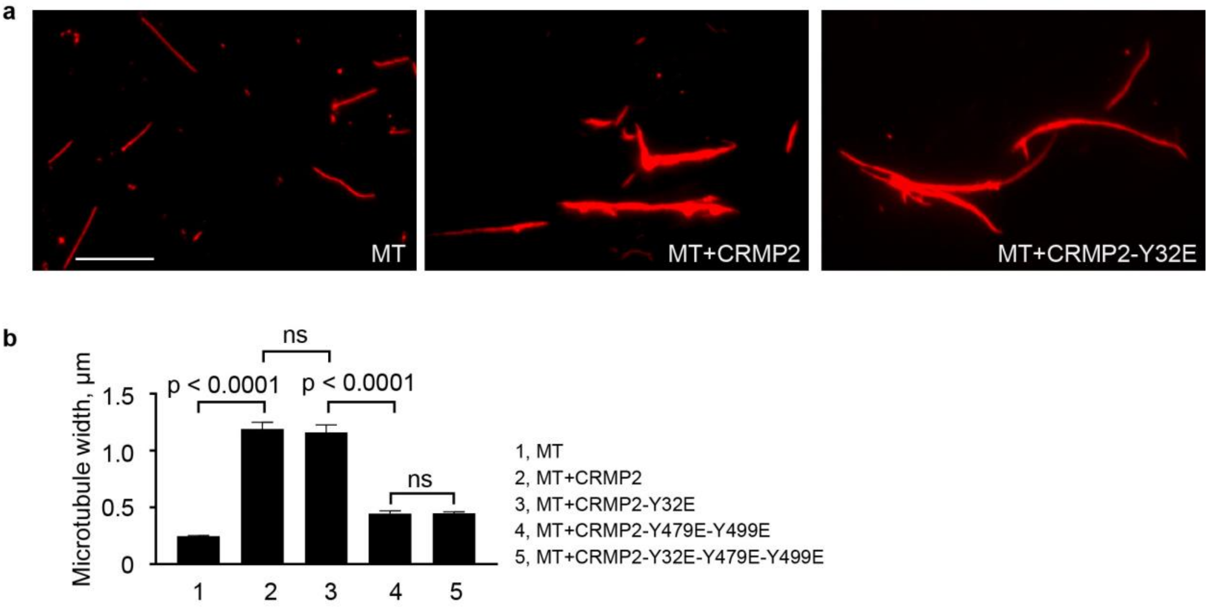

Supplementary Figure 4. (**a**) The indicated recombinant CRMP2 wild-type or CRMP2-Y32E mutant proteins were incubated with paclitaxel-stabilized rhodamine-labeled microtubules for 40 minutes at room temperature. Shown are typical results from at least three independent replicates. Scale bar is 10 µm. (**b**) Bar plots represent the mean + s.e.m. of microtubule width, from at least 150 individual microtubules per condition tested.

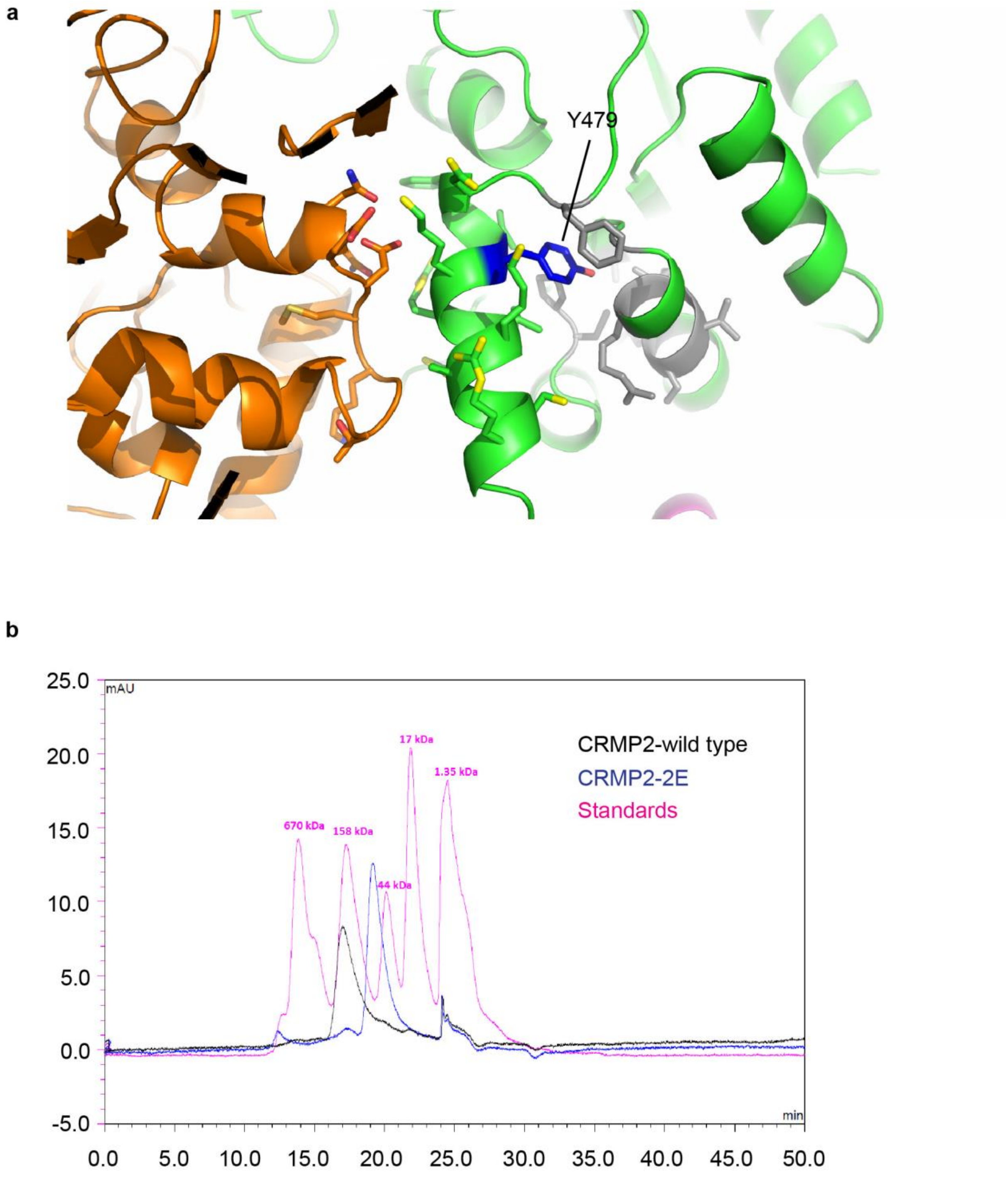

Supplementary Figure 5. (**a**) The hydrophobic pocket around Y479 (blue) is shown. The residues at tetramerization interface are shown as sticks in green (for Chain A) and orange (for Chain B). The residues in the hydrophobic pocket are shown in grey. (**b**) Analytical Gel Filtration profiles of CRMP2 wild-type (residues 13-516) and CRMP2-2E (residues 13-516). CRMP2 wild-type (shown in black) shows an elution peak around 200 kDa, corresponding to a tetramer. Phosphomimetic mutant CRMP2-2E, on the other hand, shows an elution peak around 100 kDa, corresponding to a dimer.

a

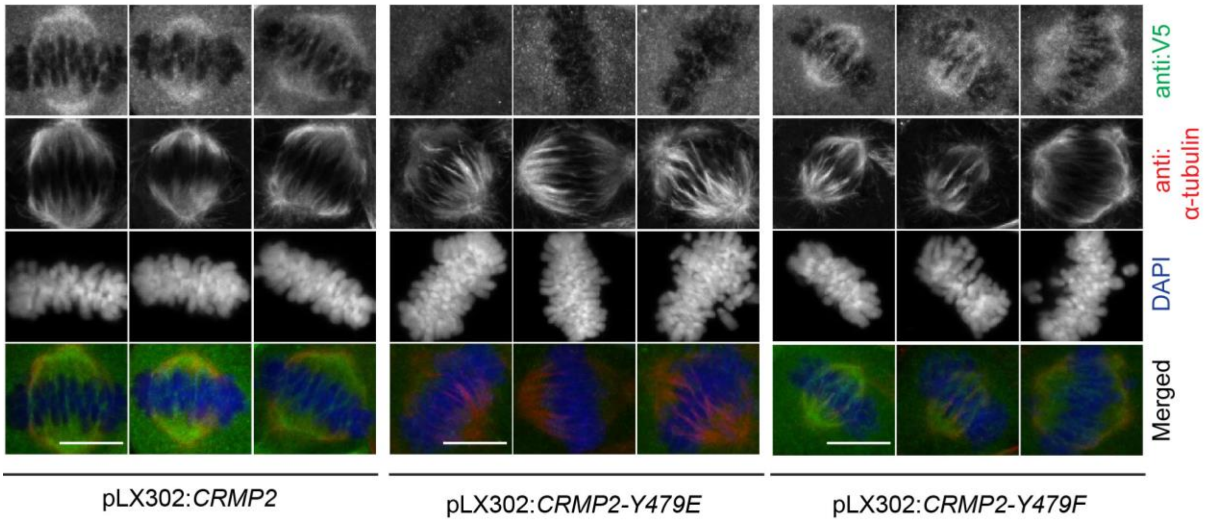

b

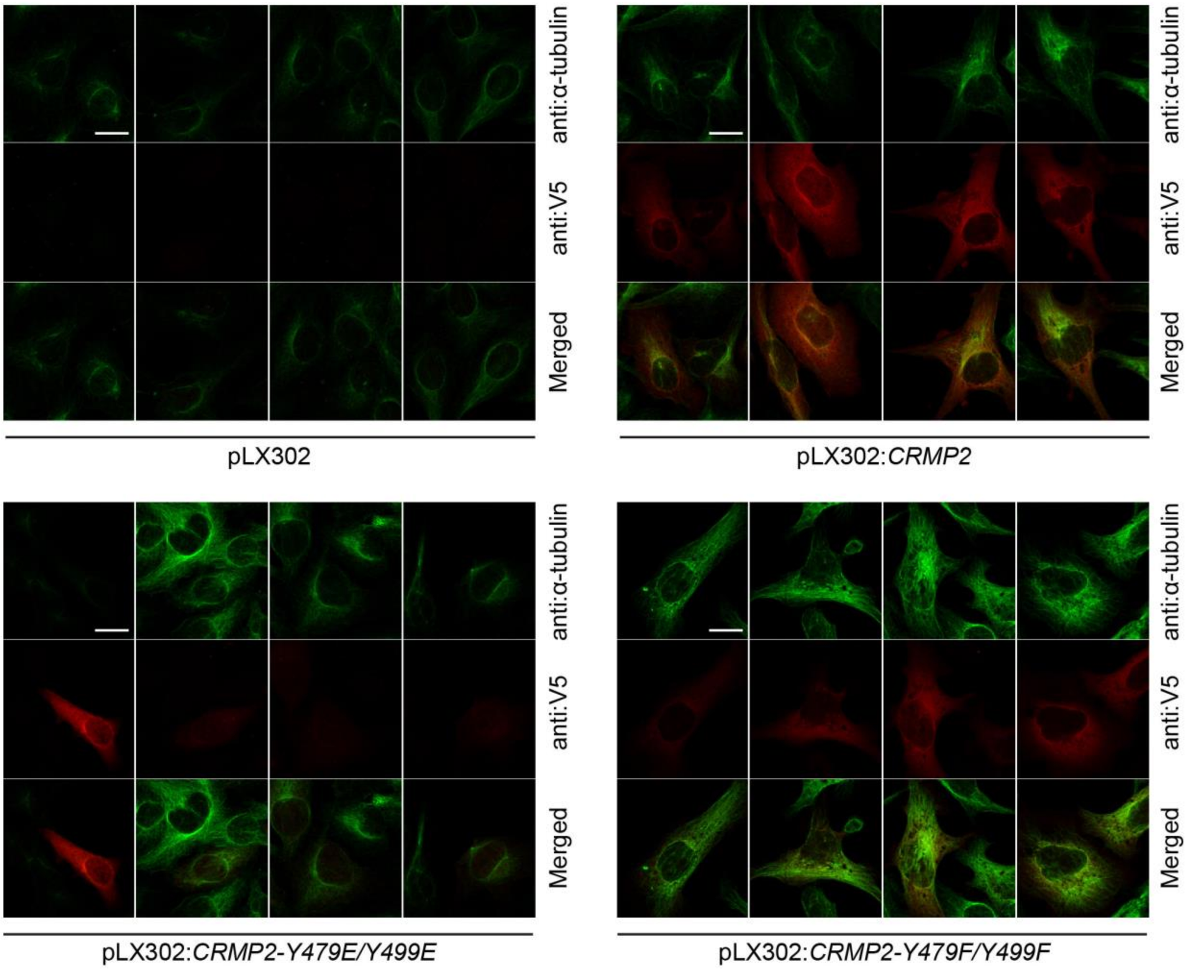

Supplementary Figure 6. (**a**) More examples of immunofluorescence staining for OVCA432 mitotic cells which were transduced with pLX302:*CRMP2*, pLX302:*CRMP2-Y479E,* or pLX302:*CRMP2-Y479F* plasmids. CRMP2, CRMP2-Y479E, or CRMP2-Y479F were detected by anti-V5 antibody. Mitotic microtubules were detected using an anti-α-tubulin

antibody. The nucleus was revealed using DAPI. Scale bar is 10 µm. (**b**) More examples of immunofluorescence staining for SKOV3 cells, which were transiently transduced with pLX302, pLX302:*CRMP2*, pLX302:*CRMP2-Y479E/Y499E*, or pLX302:*CRMP2-Y479F/Y499F* plasmids. 48 hours following transduction the cells were incubated on ice for 25 minutes before fixation and staining using the indicated antibodies to reveal microtubules (anti-α-tubulin antibody) and CRMP2 (anti-V5 antibody). Scale bar is 20 µm.

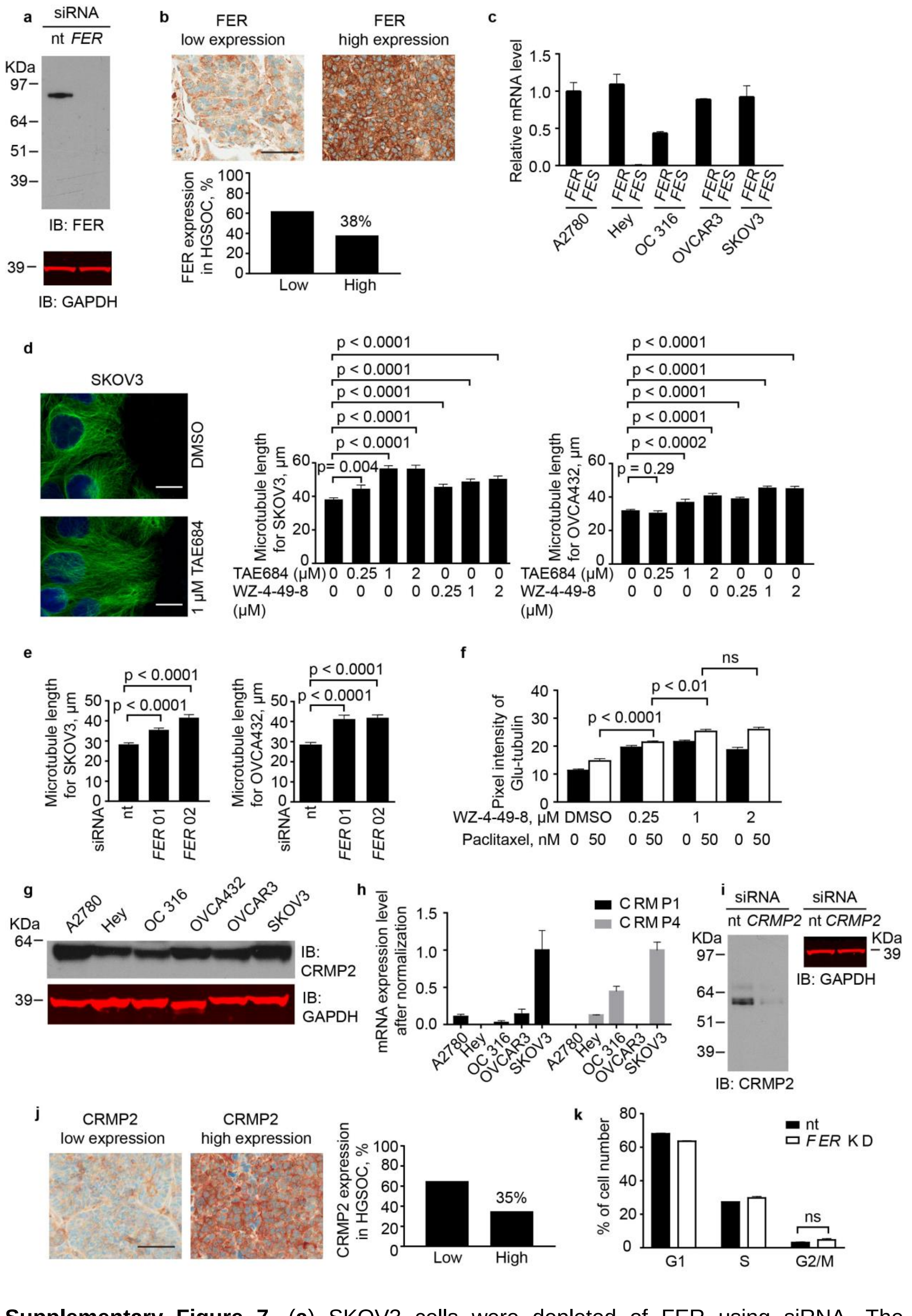

Supplementary Figure 7. (**a**) SKOV3 cells were depleted of FER using siRNA. The samples were analyzed by SDS-PAGE, and immunoblotted with the indicated antibody to

test whether this antibody detected a single band and was, therefore, suitable for estimation of FER levels using immunohistochemistry. (**b**) Representative images of FER staining in high grade serous ovarian cancer (HGSOC). Bars represent the fraction of HGSOCs that express low or high FER as indicated. (**c**) qPCR analysis for FER or FES in multiple ovarian cancer cell lines. Bar plots represent the mean + s.e.m. of relative mRNA level. (**d**) Immunofluorescence staining to measure microtubule length in SKOV3 or OVCA432 cells using anti-α-tubulin antibody. Cells were treated with increasing concentrations of FER kinase inhibitors TAE684 or WZ-4-49-8 overnight. A scratch was then performed to align the microtubules in the direction of the scratch to facilitate the measurement of the microtubule length. Cells were fixed 6 hours after scratching. Bar plots represent the mean + s.e.m. of the measured microtubule length, from at least 200 cells per condition tested. Shown are typical results from at least three independent replicates. Scale bar is 10 μm. (**e**) Immunofluorescence staining to measure microtubule length in SKOV3 or OVCA432 cells using anti-α-tubulin antibody. Cells were transfected with non-targeting (nt) siRNA, *FER* siRNA 01, or *FER* siRNA 02. Then, cell scratching and staining were performed as shown in (**d**). Bar plots represent the mean + s.e.m., from at least 200 cells per condition tested. Shown are typical results from at least three independent replicates. **(f)** Immunofluorescence staining for Glu-tubulin in SKOV3 cells, which were treated with increasing concentrations of WZ-4-49-8 overnight. Cells were treated with 50 nM paclitaxel or DMSO for 4 hours before fixation and staining. Bar plots represent mean + s.e.m. of pixel fluorescence intensity values, from at least 100 cells per condition tested, shown are typical results from at least three independent replicates. Scale bar is 10 μm. (**g**) Western blot analysis for CRMP2 expression in multiple ovarian cancer cell lines. Total lysates were analyzed by SDS-PAGE and immunoblotted using the indicated antibody. (**h**) qPCR analysis for CRMP1 or CRMP4 in multiple ovarian cancer cell lines. Bar plots represent the mean + s.e.m. of relative mRNA level. (**i**) SKOV3 cells were depleted of CRMP2 using siRNA. The samples were analyzed by SDS-PAGE, and immunoblotted with anti-CRMP2 antibody to test whether this antibody detected a predominantly single band that reduced following siRNA depletion and was, therefore, suitable for estimation of CRMP2 level using immunohistochemistry. (**j**) Representative images of CRMP2 staining in high grade serous ovarian cancer (HGSOC). Bars represent the fraction of HGSOCs by magnitude of expression of CRMP2. (**k**) SKOV3 cells were treated with non-targeting (nt) siRNA or *FER* siRNA and 96 hours later, cell cycle analysis was performed using flow cytometry. Bar plots represent the mean + s.e.m. of relative cell number (n = 3).

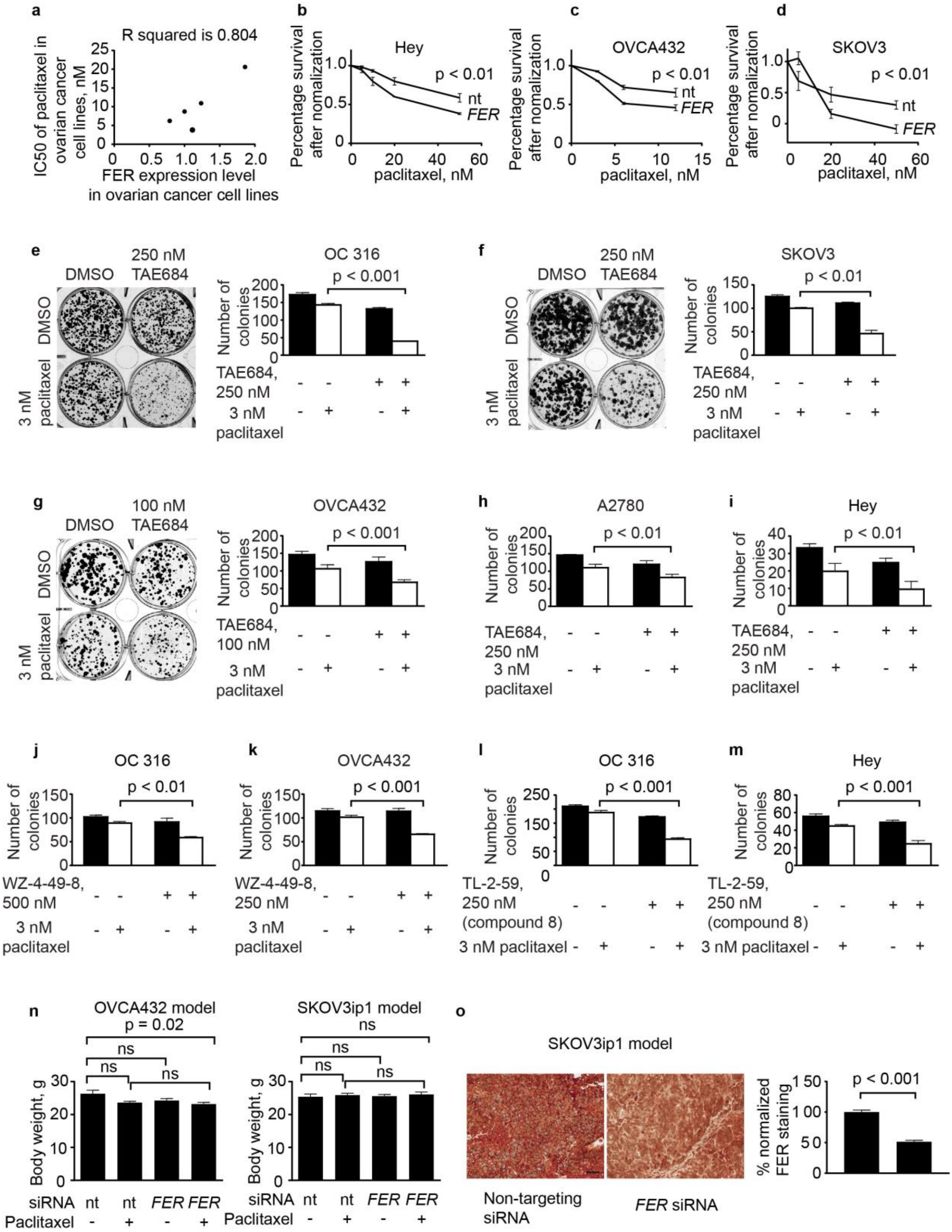

Supplementary Figure 8. (**a**) Correlation between FER protein expression level and the IC50 value of paclitaxel in multiple ovarian cancer cell lines. FER expression level was investigated at protein levels by direct blotting using anti-FER antibody. 3000 cells of the individual ovarian cancer cell lines (including A2780, Hey, OC 316, OVCAR3, and SKOV3) were plated into 96-well plates, and treated with increasing concentrations of paclitaxel after

24 hours. The cells were fixed and stained using crystal violet to estimate the number of cells 72 hours after paclitaxel treatment using OD measurements. IC50 value was produced for individual ovarian cancer cell lines using Prism 7 software. (**b**-**d**) 5000 cells of the indicated ovarian cancer cell lines were plated into individual wells of 96-well plates and transfected using non-targeting (nt) siRNAs or *FER* siRNAs. 48 hours later, the cells were treated with paclitaxel at the indicated concentrations. The cells were fixed and stained using crystal violet to estimate the number of cells 48 hours after paclitaxel treatment using OD measurements. Shown is the mean ± s.e.m. of normalized OD values from at least three independent experiments. (**e**-**i**) 500 cells of the indicated ovarian cancer cell lines were plated into individual wells of 6-well plates, and treated with either the FER inhibitor TAE684 at the indicated concentrations or diluent (DMSO). Two days later, cells were treated with 3 nM paclitaxel or diluent for 5 days before fixation and staining. Representative images of stained colonies are presented in **e-g**. Bar plots represent the mean + s.e.m. of the number of colonies from at least three independent experiments. (**j**-**k**) 500 cells of the indicated cells lines were plated into individual wells of 6-well plates, and were treated overnight with either the FER inhibitor WZ-4-49-8 or diluent (DMSO) then cells were treated with either 3 nM paclitaxel or diluent for 5 days before fixation and staining using coomassie blue. Bar plots represent the mean + s.e.m. of the number of colonies from at least three independent experiments. (**i**-**m**) 500 cells of the indicated cells lines were plated into individual wells of 6-well plates, and were treated overnight with either the FER inhibitor TL-2-59 (compound 8) or diluent (DMSO) then cells were treated with either 3 nM paclitaxel or diluent for 5 days before fixation and staining using coomassie blue. Bar plots represent the mean + s.e.m. of the number of colonies from at least three independent experiments. (**n**) Quantification analysis of the mouse body weight in the two xenograft models of ovarian cancer (OVCA432 and SKOV3ip1 ovarian cancer cells). Bar plots represent the mean + s.e.m.. (**o**) Representative images of FER immunohistochemical staining for SKOV3ip1 xenograft model that were treated with non-targeting (nt) siRNA or *FER*-targeting siRNA. Bar plots represent the mean + s.e.m. for the quantification analysis of FER protein staining.

Figure 2c, top panel
in the left column

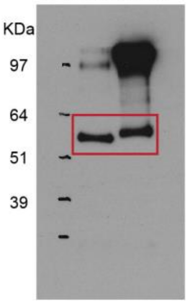

Figure 2c, middle panel
in the left column

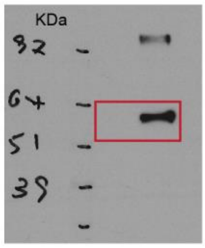

Figure 2c, bottom panel
in the left column

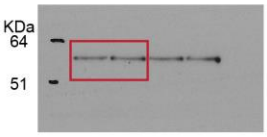

Figure 2c, top panel
in the middle column

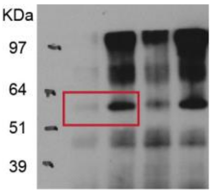

Figure 2c, middle panel
in the middle column

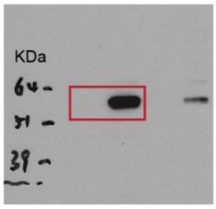

Figure 2c, bottom panel
in the middle column

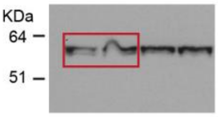

Figure 2c, top panel
in the right column

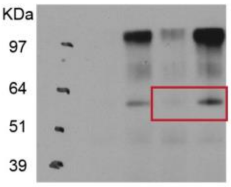

Figure 2c, middle panel
in the right column

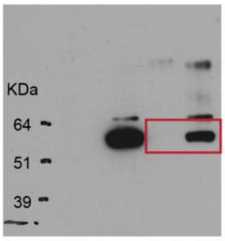

Figure 2c, botom panel
in the right column

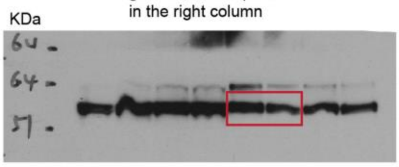

Figure 2d, top panel
in the left column

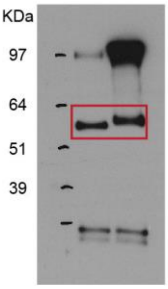

Figure 2d, middle panel
in the left column

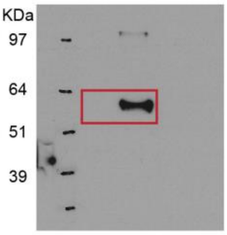

Figure 2d, bottom panel
in the left column

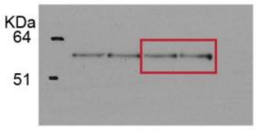

Figure 2d, top panel
in the middle column

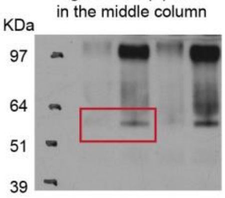

Figure 2d, middle panel
in the middle column

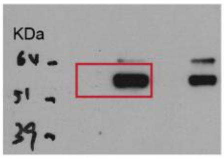

Figure 2d, bottom panel
in the middle column

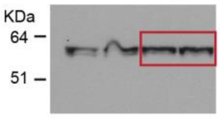

Figure 2d, top panel
in the right column

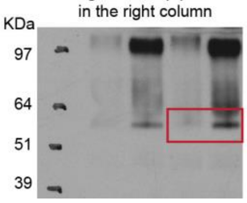

Figure 2d, middle panel
in the right column

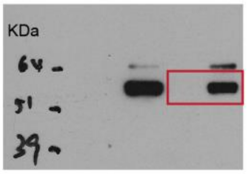

Figure 2d, bottom panel
in the right column

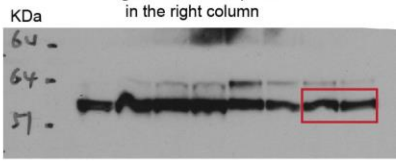

Figure 2e, first panel

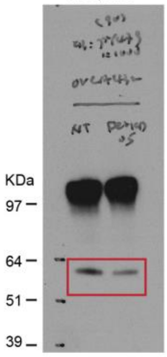

Figure 2e, second panel

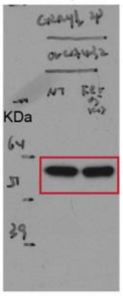

Figure 2e, third panel

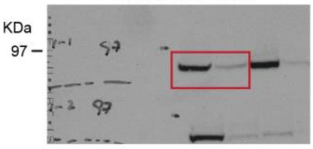

Figure 2e, fourth panel

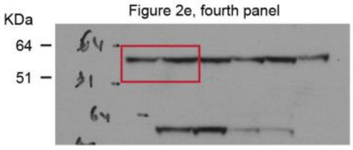

Figure 2e, fifth panel

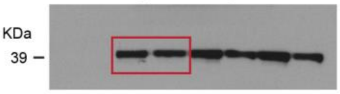

Figure 5a, top panel

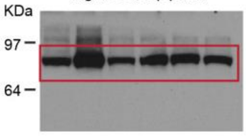

Figure 5a, bottom panel

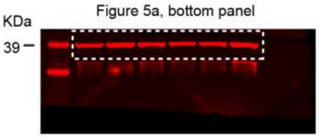

Figure 5b, top panel

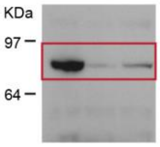

Figure 5b, bottom panel

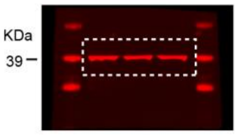

Supplementary Figure 1a, top panel

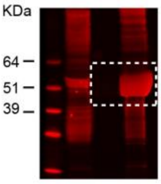

Supplementary Figure 1a, middle panel

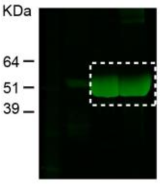

Supplementary Figure 1a, bottom panel

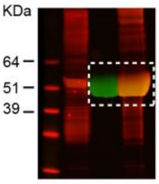

Supplementary Figure 1b, left panel

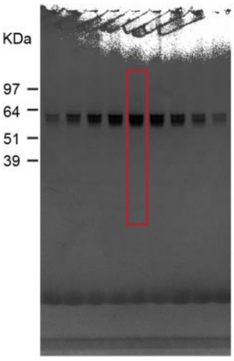

6His-CRMP2

Supplementary Figure 1b, right panel

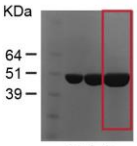

Tubulin

Supplementary Figure 3a, top panel in the left column

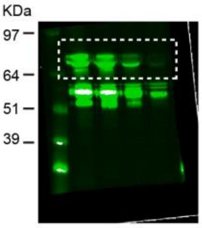

Supplementary Figure 3a, middle panel in the left column

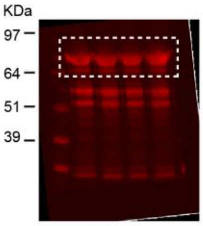

Supplementary Figure 3a, bottom panel in the left column

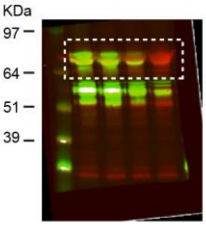

Supplementary Figure 3a, top panel in the right column

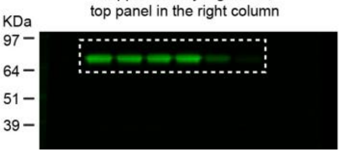

Supplementary Figure 3a, middle panel in the right column

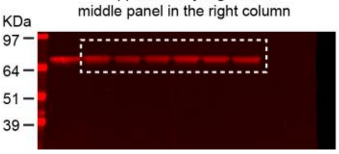

Supplementary Figure 3a, bottom panel in the right column

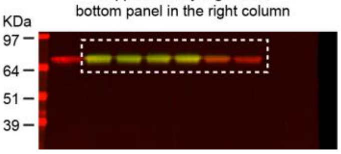

Supplementary Figure 3b, top panel

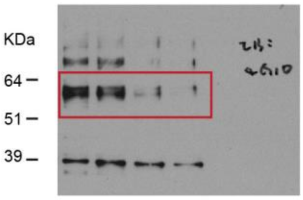

Supplementary Figure 3b, middle panel

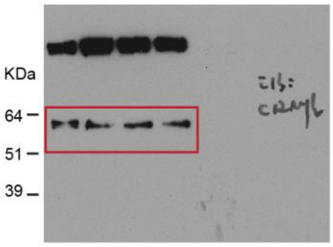

Supplementary Figure 3b, bottom panel

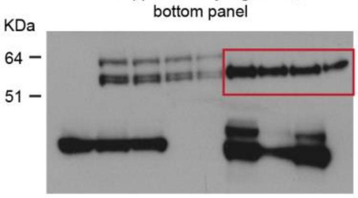

Supplementary Figure 3d, top panel

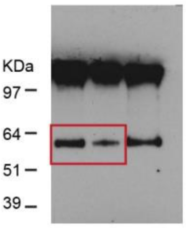

Supplementary Figure 3d, middle panel

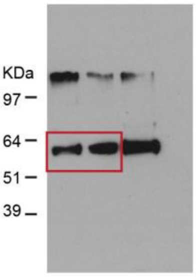

Supplementary Figure 3d, bottom panel

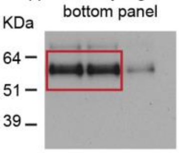

Supplementary Figure 3e, first panel

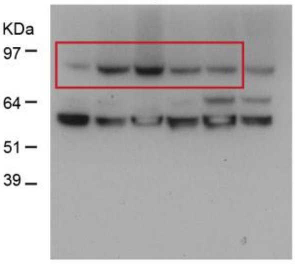

Supplementary Figure 3e, third panel

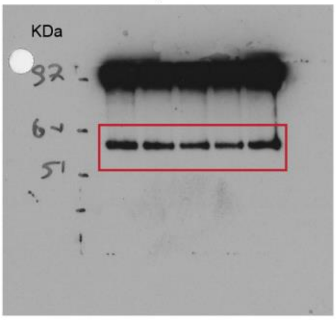

Supplementary Figure 3e, fourth panel

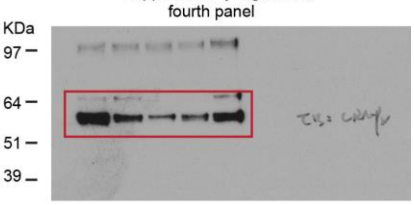

Supplementary Figure 3e, second panel

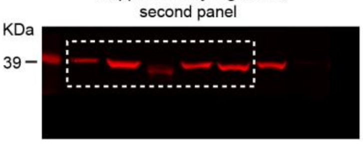

Supplementary Figure 3g, top panel

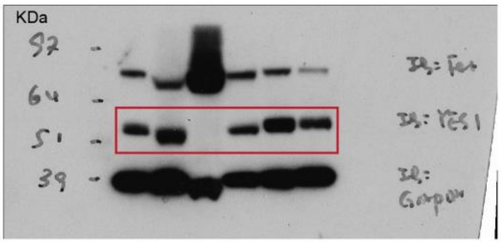

Supplementary Figure 3g, bottom panel

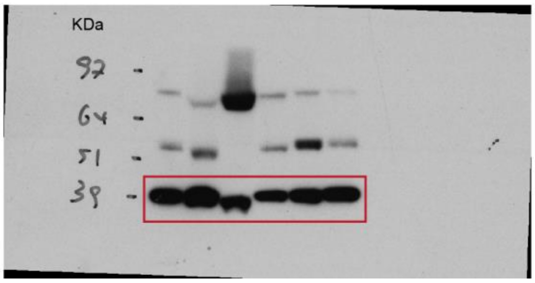

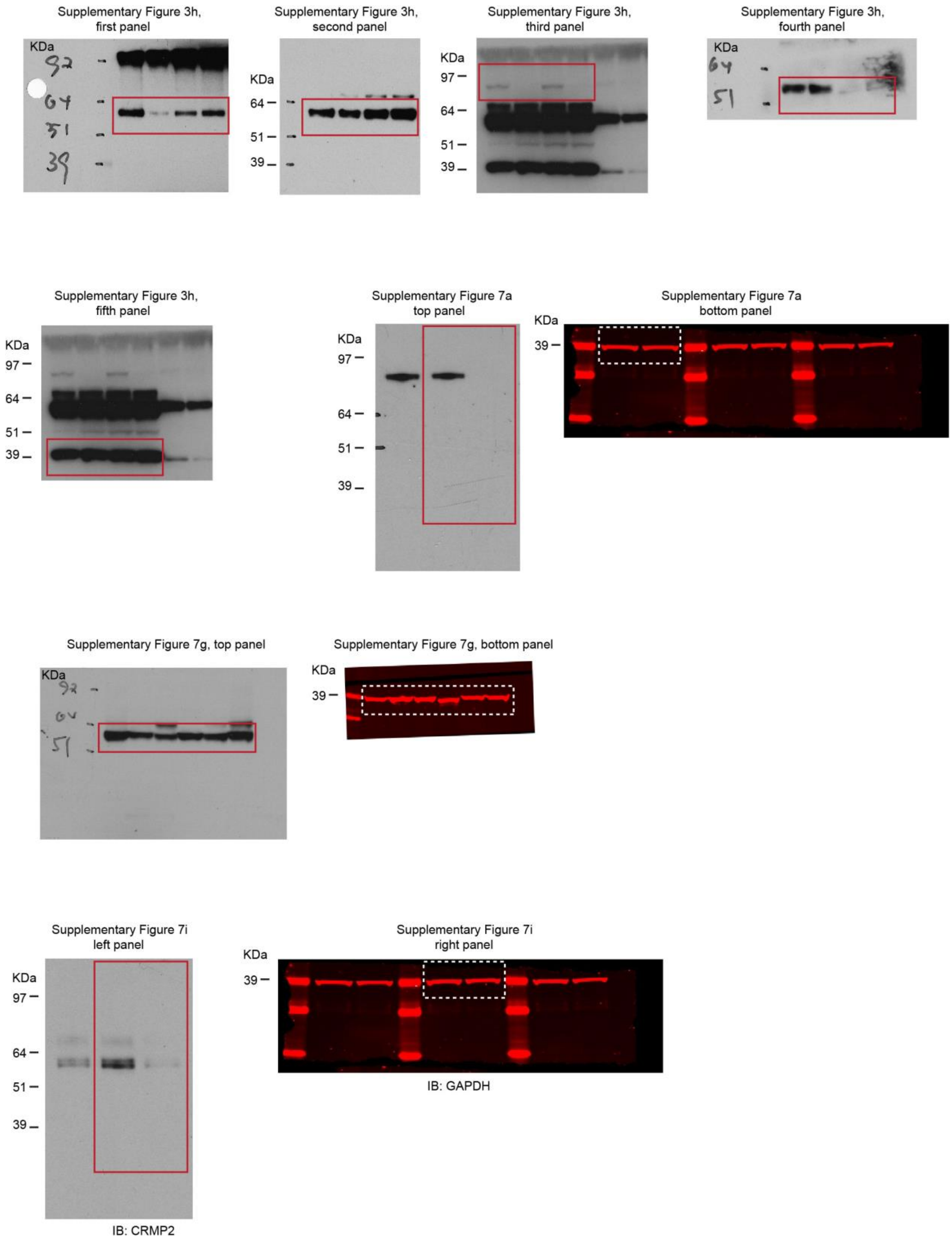

Supplementary Figure 9. Uncropped blots and gels used in the main and supplementary figures.

a

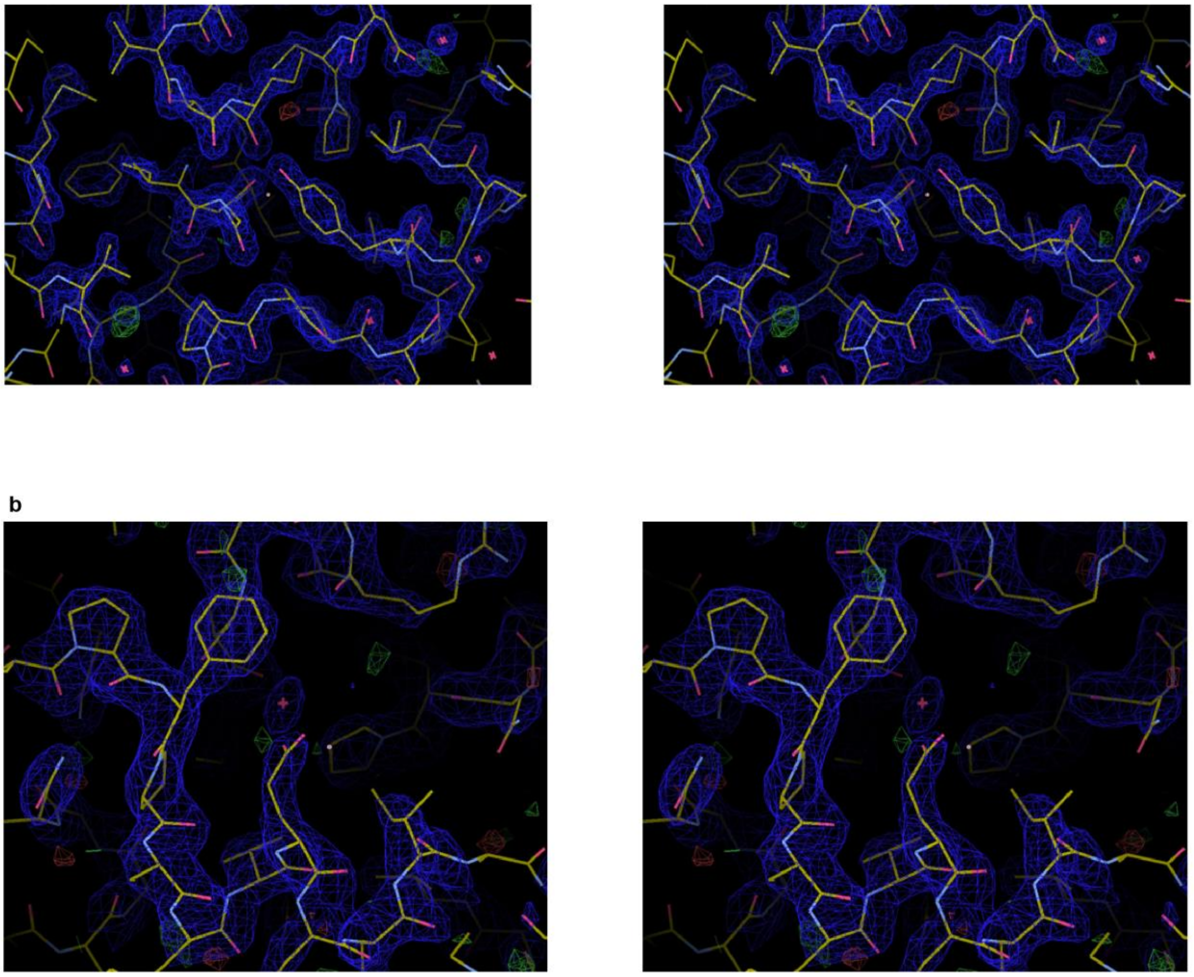

Supplementary Figure 10. A stereo image of a portion of the electron density map for crystallographic structure of CRMP2-WT (a) or CRMP2-2E (b).

Supplementary Table 1. Primers used for construction in this study are listed.

Primer name	Description	Primer sequence (from 5' to 3')
CRMP2 1F	primer used to clone CRMP2:pGEX-KG (residues 1-516) for GST-CRMP2 (residues 1-516) protein expression	GTCCCGGGTATGTCTTATCAGGGGAAGAAAAATATT
CRMP2 1R	primer used to clone CRMP2:pGEX-KG (residues 1-516) for GST-CRMP2 (residues 1-516) protein expression	GTTCTAGATCAGGCTGGAGTGACTGTCTT
CRMP2 2F	primer used to clone CRMP2:pNIC Bsa4 for 6His-CRMP2 protein expression	TACTTCCAATCCATGATGTCTTATCAGGGGAAGAAA
CRMP2 2R	primer used to clone CRMP2:pNIC Bsa4 for 6His-CRMP2 protein expression	TATCCACCTTTACTGCTAGCCCAGGCTGGTGATGTT
CRMP2 3F	primer used to clone CRMP2:pNIC Bsa4 (residues 13-516) for 6His-CRMP2 (residues 13-516) protein expression	TACTTCCAATCCATGACGAGCGATCGTCTTCTGAT
CRMP2 3R	primer used to clone CRMP2:pNIC Bsa4 (residues 13-516) for 6His-CRMP2 (residues 13-516) protein expression	TATCCACCTTTACTGTCAGGCTGGAGTGACTGTCTTGG
CRMP2 4F	primer used for CRMP2-Y479E mutation	TTCCCTGATTTTGTTGAAAAGCGTATCAAGGCA
CRMP2 4R	primer used for CRMP2-Y479E mutation	TGCCTTGATACGCTTTTCAACAAAATCAGGGAA
CRMP2 5F	primer used for CRMP2-Y479F mutation	TTCCCTGATTTTGTTTTCAAGCGTATCAAGGCA
CRMP2 5R	primer used for CRMP2-Y479F mutation	TGCCTTGATACGCTTGAAAACAAAATCAGGGAA
CRMP2 6F	primer used for CRMP2-Y499E mutation	CCTCGTGGCCTGGAGGACGGACCT
CRMP2 6R	primer used for CRMP2-Y499E mutation	AGGTCCGTCCTCCAGGCCACGAGG
CRMP2 7F	primer used for CRMP2-Y499F mutation	CCTCGTGGCCTGTTTGACGGACCT
CRMP2 7R	primer used for CRMP2-Y499F mutation	AGGTCCGTCAAACAGGCCACGAGG
CRMP2 8F	primer used for CRMP2-Y32F mutation	TGACCAGTCGTTCTTTGCAGACATATA
CRMP2 8R	primer used for CRMP2-Y32F mutation	TATATGTCTGCAAAGAACGACTGGTCA
CRMP2 9F	primer used for CRMP2-Y251F mutation	CAACTGCCCGCTGTTTATCACCAAGGT
CRMP2 9R	primer used for CRMP2-Y251F mutation	ACCTTGGTGATAAACAGCGGGCAGTTG
CRMP2 10F	primer used for CRMP2-Y275F mutation	GGGAACTGTGGTGTTTGGCGAGCCCAT
CRMP2 10R	primer used for CRMP2-Y275F mutation	ATGGGCTCGCCAAACACCACAGTTCCC
CRMP2 11F	primer used for CRMP2-Y431F mutation	CAGCTCTCTCGAGTTCAACATCTTTGA
CRMP2 11R	primer used for CRMP2-Y431F mutation	TCAAAGATGTTGAACTCGAGAGAGCTG
CRMP2 12 F	primer used to clone CRMP2:pLX302	GGGGACAAGTTTGTACAAAAAAGCAGGCTTCGAAGGAGATAGAACC ATGTCTTATCAGGGGAAGAAAAAT
CRMP2 12 R	primer used to clone CRMP2:pLX302	GGGGACCACTTTGTACAAGAAAGCTGGGTCGCCCAGGCTGGTGATG TTGGC

Supplementary Table 2. The primer sequences for qPCR are listed.

Primer name	Description	Primer sequence (from 5’ to 3’)
FES 1F (qPCR)	to determine human FES mRNA level	TCAAGGCCAAGTTTCTACAGG
FES 1R (qPCR)	to determine human FES mRNA level	GAGGAAGGTCAGGAAGTCG
FER 1F (qPCR)	to determine human FER mRNA level	TGTCTCGTCAAGAGGATGGTG
FER 1R (qPCR)	to determine human FER mRNA level	CAAACCCCTAAGCTGAAGGTC
GAPDH 1F (qPCR)	to determine human GAPDH mRNA level	GCAAATTCCATGGCACCG
GAPDH 1R (qPCR)	to determine human GAPDH mRNA level	TCGCCCCACTTGATTTTGG

Supplementary Table 3. Shown are the information and dilutions of commercial primary antibodies used in this study. WB, western blot; IF, immunofluorescence staining; IP, immunoprecipitation; IHC, immunohistochemistry.

**Antibodies**	**SOURCE**	**Catalogue number**
Mouse anti-α-tubulin (IF, 1:200)	Sigma-Aldrich	Cat# T5168
Rabbit anti-CRMP2 (IF, 1:200; IHC, 1:200; IP, 1:100; WB, 1:3000)	Abcam	Cat# ab129082
Rabbit anti-detyrosinated tubulin (Glu-tubulin) (IF, 1:100)	Millipore	Cat# AB3201
Mouse anti-FER (IHC, 1:300)	Abcam	Cat# ab130199
Rabbit anti-FER (WB, 1:5000)	Abcam	Cat# ab52479
Rabbit anti-YES (WB, 1:1000)	Abcam	Cat# ab133314
Mouse anti-GAPDH (WB, 1:5000)	Proteintech	Cat# 60004-1
Mouse anti-GST (WB, 1:5000)	Cell Signaling	Cat# 2624S
Mouse anti-His (WB, 1:2000)	Cell Signaling	Cat# 2366S
Mouse anti-Phosphotyrosine (4G10) (WB, 1:1000)	Millipore	Cat# 05-321
Rabbit anti-V5 (IF, 1:200)	Abcam	Cat# ab9116

Supplementary Table 4. The data collection and refinement statistics for protein structure determination.

	CRMP2 (residues 13-516) (PDB ID 5MKV)	CRMP2 (residues 13-516)-2E mutant (PDB ID 5MLE)
Wavelength (Å)	0.92	0.92
Resolution range (Å)	19.87 - 1.8 (1.86 - 1.8)	83.98 - 2.48 (2.56 - 2.48)
Space group	P 1 21 1	C 2 2 21
Unit cell dimensions	79.90 157.51 87.42<br>90 93.61 90	70.90 185.82 196.31<br>90 90 90
Total reflections	695101 (69999)	313582 (31996)
Unique reflections	194435 (19419)	46370 (4564)
Multiplicity	3.6 (3.6)	6.8 (7.0)
Completeness (%)	97.7 (97.8)	99.9 (99.9)
Mean I/sigma(I)	15.8 (3.0)	6.2 (1.0)
Wilson B-factor ($Å^2$)	21.9	36.1
R-merge	0.051 (0.372)	0.319 (1.826)
R-meas	0.060 (0.436)	0.345 (1.973)
R-pim	0.031 (0.227)	0.132 (0.742)
CC1/2 (%)	99.0 (87.0)	98.0 (57.0)
Reflections used in refinement	194312 (19419)	46359 (4564)
Reflections used for R-free	9714 (971)	2328 (231)
R-work (%)	17.35 (22.89)	21.60 (36.14)
R-free (%)	20.31 (27.48)	25.54 (38.84)
CC (work)	0.96 (0.80)	0.92 (0.50)
CC (free)	0.94 (0.76)	0.87 (0.33)
Number of non-hydrogen atoms	15644	7459
macromolecules	14960	7221
ligands	88	63
solvent	596	175
Protein residues	1949	940
RMS (bonds) (Å)	0.020	0.014
RMS (angles) (^{o})	1.9	1.6
Ramachandran favored (%)	96.85	95.07
Ramachandran allowed (%)	2.99	4.28
Ramachandran outliers (%)	0.15	0.64
Rotamer outliers (%)	1.6	4.83
Molprobity Clashscore	3.88	3.11
Molprobity Percentile	98th	100th
Average B-factor ($Å^2$)	25.55	40.72
macromolecules	25.45	40.68
ligands	41.81	70.05
solvent	25.69	31.83
