# Supplementary material for: Tuning microtubule dynamics to enhance cancer therapy by modulating FER-mediated CRMP2 phosphorylation
Source: Nat Commun. 2018 Feb 2;9:476. doi: 10.1038/s41467-017-02811-7 (PMC5797184; doi:10.1038/s41467-017-02811-7)
Supplement: Supplementary file 3 — Description of Additional Supplementary Files [file 41467_2017_2811_MOESM3_ESM.pdf]

Description of Additional Supplementary Files

File Name: Supplementary Movie 1
Description: Time series movie of microtubule elongation, related to the top panel of Supplementary Figure 1d.

File Name: Supplementary Movie 2
Description: Time series movie of microtubule elongation in the presence of CRMP2, related to the middle panel of Supplementary Figure 1d.

File Name: Supplementary Movie 3
Description: Time series movie of microtubule elongation in the presence of pCRMP2 (phospho-CRMP2), related to the bottom panel of Supplementary Figure 1d.
